# Design of Turmeric Rhizome Extract Nano-Formula for Delivery to Cancer Cells

**DOI:** 10.3390/molecules27030896

**Published:** 2022-01-28

**Authors:** Sakchai Auychaipornlert, Pojawon Prayurnprohm Lawanprasert, Suchada Piriyaprasarth, Pongtip Sithisarn, Supachoke Mangmool

**Affiliations:** 1 Department of Manufacturing Pharmacy, Faculty of Pharmacy, Mahidol University, Bangkok 10400, Thailand; asakchai.smc@gmail.com; 2 Department of Pharmaceutical Technology, Faculty of Pharmacy, Silpakorn University, Nakhon Pathom 73000, Thailand; PIRIYAPRASARTH_S@silpakorn.edu; 3 Department of Pharmacognosy, Faculty of Pharmacy, Mahidol University, Bangkok 10400, Thailand; pongtip.sit@mahidol.ac.th; 4 Department of Pharmacology, Faculty of Science, Mahidol University, Bangkok 10400, Thailand; supachoke.man@mahidol.ac.th

**Keywords:** turmeric rhizome extract, turmeric oil, curcumin, HepG2, nanoparticles, anticancer

## Abstract

Novel turmeric rhizome extract nanoparticles (TE-NPs) were developed from fractions of dried turmeric (*Curcuma longa* Linn.) rhizome. Phytochemical studies, by using HPLC and TLC, of the fractions obtained from ethanol extraction and solvent–solvent extraction showed that turmeric rhizome ethanol extract (EV) and chloroform fraction (CF) were composed mainly of three curcuminoids and turmeric oil. Hexane fraction (HE) was composed mainly of turmeric oil while ethyl acetate fraction (EA) was composed mainly of three curcuminoids. The optimal TE-NPs formulation with particle size of 159.6 ± 1.7 nm and curcumin content of 357.48 ± 8.39 µM was successfully developed from 47-run D-optimal mixture–process variables experimental design. Three regression models of z-average, d_50_, and d_90_ could be developed with a reasonable accuracy of prediction (predicted r^2^ values were in the range of 0.9120–0.9992). An in vitro cytotoxicity study using MTT assay demonstrated that the optimal TE-NPs remarkably exhibited the higher cytotoxic effect on human hepatoma cells, HepG2, when compared with free curcumin. This study is the first to report nanoparticles prepared from turmeric rhizome extract and their cytotoxic activity to hepatic cancer cells compared with pure curcumin. These nanoparticles might serve as a potential delivery system for cancer therapy.

## 1. Introduction

Turmeric is a dried rhizome of Curcuma longa Linn. of the family Zingiberaceae. It is mostly cultivated in Southern and Southeast Asia [1]. A number of pharmacological activities, especially anticancer activities, of compounds contained in turmeric were reported [2]. Most of them showed the pharmacological activities of curcumin, the major active compound found in the turmeric rhizome [2,3,4,5]. Anticancer activities of its analog compounds and turmeric oil were also reported [6,7,8,9]. Turmeric and curcumin can be considered as safe [10,11]. However, the low aqueous solubility and poor stability of curcumin led to limitations of its use as a therapeutic agent. Many advanced technologies were proposed to overcome this limitation [12,13,14].

Nanotechnology had been the one potential strategy for treatment of cancer diseases [15,16,17,18,19]. The nanoscale materials are currently being investigated to improve their specificity towards cancer cells and towards subcellular compartments in order to reduce systemic toxicity [15,16,17,18,19,20]. Curcumin has also been developed in nanoscale [21,22,23,24,25,26,27,28,29,30,31,32,33,34,35,36,37]. For example, Shaikh and coworkers (2009) [29] prepared curcumin-loaded poly(lactide-co-glycolide) (curcumin loaded PLGA) nanoparticles by using an emulsion–diffusion–evaporation method. Curcumin-loaded PLGA nanoparticles demonstrated at least 9-fold increase in oral bioavailability when compared with curcumin administered with piperine as an absorption enhancer. Anand and colleagues (2010) [21] prepared curcumin-loaded PLGA-PEG nanoparticles by a nanoprecipitation technique. In vitro study showed that curcumin nanoparticles exhibited rapid cellular uptake and induced apoptosis in human chronic myeloid leukemia (KBM-5). Additionally, curcumin nanoparticles could inhibit cell proliferation of various tumor cells, i.e., human leukemia (KBM-5 and Jurkat), prostate (DU145), breast (MDA-MB-231), colon (HCT116), and esophageal (SEG-1) cancer cells. Mohanty C. and Coworkers (2010) [27] prepared curcumin nanoparticles by an emulsifying method with a group of surfactants, i.e., glycerol monooleate (GMO), polyvinyl alcohol (PVA), and Pluronic^®^. These curcumin nanoparticles were more effective than curcumin against different cancer cells. In addition, Zhao L and coworkers (2012) [37] prepared curcumin-loaded mixed micelles (Cur-PF) that were composed of Pluronic P123 and Pluronic F68. They found that Cur-PF presented a sustained release property. O/W nanoemulsion containing curcumin was prepared by using high-speed and high-pressure homogenization [34]. Medium chain triacylglycerols (MCT) and Tween 20 were used as oil phase and emulsifier, respectively. This 1% curcumin o/w nanoemulsion exhibited an inhibition effect of 12-O-tetradecanoyl- horbol-13-acetate (TPA)-induced edema of mouse ear.

The enhancements of anticancer activities were found when nanoparticles of an anticancer drug were coated with hyaluronic acid [38,39,40]. This may be because the high binding affinity of hyaluronic acid to the CD44 receptor, which overexpresses in tumor cell [39,41,42,43].

The spontaneous nanoemulsion formed by the solvent displacement method called the Ouzo effect was originally found in anise-flavored alcoholic beverages [44,45,46,47,48,49,50,51,52,53,54,55]. Vitale and Katz explained that the effect occurs when solutions are rapidly brought into the metastable region by the addition of water. When the solubility of some of solutes decreases more rapidly, supersaturation is then large, and homogeneous nuclei form spontaneously [47].

A number of studies in the literature have focused on the nanoparticle formation of curcumin. The study of nanoparticle formation from turmeric rhizome extract has not yet been reported. In this study, we aimed to develop nanoparticles from various turmeric rhizome fractions by using the solvent displacement method and investigated the cytotoxic activity of the obtained turmeric rhizome extract nanoparticles toward HepG2 cells.

## 2. Results and Discussion

### 2.1. Curcuminoids Content of Turmeric Rhizome Fractions

**Figure 1:**
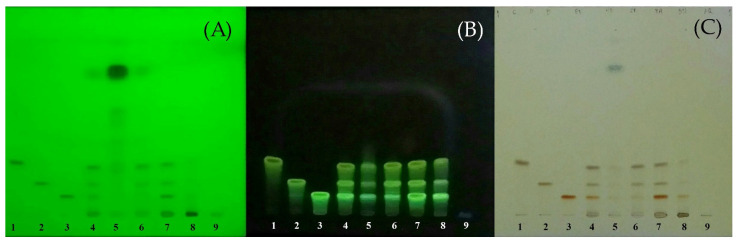
TLC chromatograms of standard curcuminoids and all turmeric rhizome fractions. Track 1 = standard curcumin (CM); 2 = standard desmethoxycurcumin (DCM); 3 = standard bisdesmethoxycurcumin; 4 = EV; 5 = HE; 6 = CF; 7 = EA; 8 = BU; 9 = AQ. Solvent system, benzene/chloroform/ethanol (49:49:2 by volume). Detection: (**A**) = UV 254 nm; (**B**) = UV 366 nm; (**C**) = 10% phosphomolybdic spray reagent; all heated at 105 °C for 5 min, detected under white light.

The chromatograms of turmeric rhizome fractions analyzed by thin layer chromatography (TLC) detected by ultraviolet (UV) and spray reagent are shown in Figure 1. It was found that turmeric rhizome fractions except aqueous fraction (AQ) (track 9) developed chromatographic bands with hRf values corresponding to the standard three curcuminoids (Figure 1). The hRf values of standards, curcumin (CM, track 1), desmethoxycurcumin (DCM, track 2), and bisdesmethoxycurcumin (BDCM, track 3) determined by spraying with 10% phosphomolybdic spray reagent were 18.13, 16.88, and 10.00, respectively. In addition, the turmeric rhizome ethanol extract (EV), hexane fraction (HE), and chloroform fraction (CF) developed blue bands above three bands of standard curcuminoids at hRf values of 78.75, 79.38, and 80.00, respectively. This blue band was clearly seen for HE detected under UV 254 nm and 10% phosphomolybdic spray reagent (track 5). The remaining two bands of EV (track 4) and CF (track 6) showed pale blue bands under UV 254. The band at an hRf value of approximately 80 might be dl-turmerone, one of turmeric oil’s components, as specified in the Thai Herbal Pharmacopoeia 2020 vol 1 [56].

**Figure 2:**
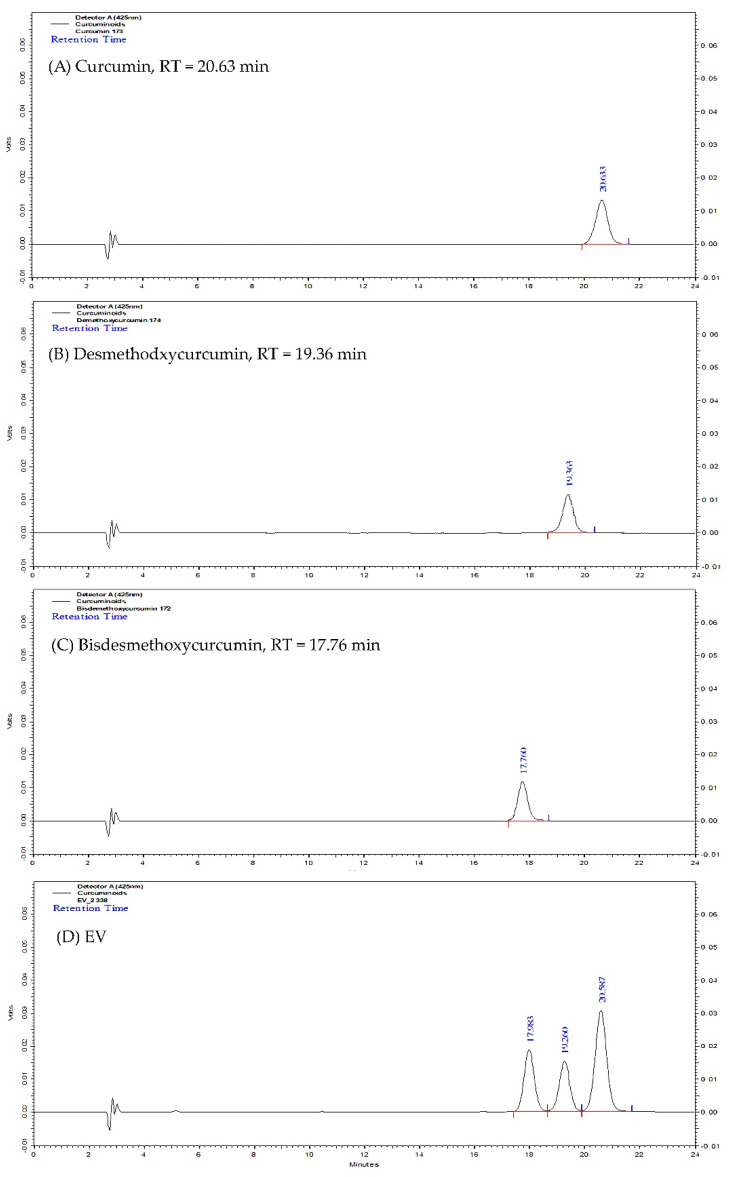
HPLC fingerprint of standard curcumin (**A**); standard desmethoxycurcumin (**B**); standard bisdesmethoxycurcumin (**C**); and turmeric rhizome ethanol extract (EV, (**D**)).

Turmeric rhizome fractions were also analyzed by the validated modified high performance liquid chromatographic method (HPLC) [57]. The HPLC fingerprints of the standards and turmeric rhizome fractions are shown in Figure 2. For ethanol extract (EV), three peaks that have retention time corresponding to standard curcumin, desmethoxycurcumin, and bisdesmethoxycurcumin at 20.63, 19.36, and 17.60 min, respectively, are shown. Similar results were found for the other turmeric rhizome fractions except AQ, i.e., hexane fraction (HE), chloroform fraction (CF), ethyl acetate fraction (EA), and n-butanol fraction (BU). The quantitative analysis results of turmeric rhizome fractions are shown in Table 1. The results show that all turmeric rhizome fractions except AQ contained various amounts of three main curcuminoids. The highest total curcuminoid content was found in EA fraction (421.41 mg/g of dried extract), which contained bisdesmethoxycurcumin (BDCM) as the major component. Low curcuminoid content was found in HE and BU fractions (4.88 and 12.14 mg/g of dried extract, respectively). TLC and HPLC analysis suggested that the phytochemical profile of turmeric rhizome fractions prepared in this study were mainly composed of curcuminoids and turmeric oil. This result is consistent with the results reported previously [56,58,59,60,61].

### 2.2. Determination of the Optimal Turmeric Rhizome Extract Nanoparticles Formulation

The curcuminoids content and particle characteristic results of 47 designed TE-NP formulations are shown in Table S1 (Supplementary Materials).

The significant regression model for dependent variables (*p*-value < 0.05) with a high degree of model fitness (r^2^ = 0.8961–0.9635) were obtained for curcumin content and %LA, z-average, d_50_, and d_90_, defined as Y_m1_, Y_m2_, Y_m5_, Y_m6_, and Y_m7_. This indicates that these regression models have power to explain the effect of independent variables on the dependent variables of TE-NPs. The regression models for curcumin analysis created in this work were the combined reduced quadratic × linear models. For particle analysis, the combined reduced quadratic × 2FI, quadratic × linear, and linear × 2FI models were obtained for z-average, d_50_, and d_90_, respectively.

The regression model equations in terms of actual components and actual factors of all dependent variables were obtained as follows:

(1) $$\sqrt{Y_{m1}}=0.072A\;+5.218B\;+4.520C\;+0.825\mathrm{AB}\;+1.930\mathrm{AC}\;+85.035\mathrm{AD}$$

(2) $$\begin{array}{cc} \sqrt{Y_{m2}}=0.155A & \;+3.576B\;+2.914C\;+1.699\mathrm{AB}\;+2.883\mathrm{AC}\;+0.174\mathrm{AD}\\ & \;+0.301\mathrm{BC}\;-197.093\mathrm{BD}\;-0.164\mathrm{BE}\;-29.685\mathrm{CD}\\ & \;+0.039\mathrm{CE}\;-0.388\mathrm{ABE}-0.311\mathrm{ACE}\;+219.773\mathrm{BCD} \end{array}$$

(3) $$\begin{array}{cc} Y_{m5}=70.592A & +61.265B\;+62.690C\;-2.196\mathrm{AB}\;-10.592\mathrm{AC}\;+9.530\mathrm{AE}\\ & -7.599\mathrm{BC}\;+242.050\mathrm{BD}\;+2.246\mathrm{BE}\;+3.625\mathrm{CE}\;\\ & +3.484\mathrm{ABE}\;+742.110\mathrm{BDE} \end{array}$$

(4) $$\begin{array}{cc} \sqrt{Y_{m6}}=5.443A & +4.603B\;+4.962C\;-0.582\mathrm{AC}\;+0.828\mathrm{AE}\;+102.780\mathrm{BD}\;\\ & +0.520\mathrm{BE}\;+0.380\mathrm{CE} \end{array}$$

(5) $$\begin{array}{cc} \sqrt{Y_{m7}}=7.128A & +6.324B\;+6.273C\;+1.402\mathrm{AE}\;-12.783\mathrm{BD}\;+0.443\mathrm{BE}\;\\ & +1.008\mathrm{CE}\;+154.056\;\mathrm{BDE} \end{array}$$

where Y_m1_ = curcumin content (µM), Y_m2_ = % label amount of curcumin (%LA), Y_m5_ = z-average (nm), Y_m6_ = d_50_ (nm), Y_m7_ = d_90_ (nm), A = HE (%*w*/*w*), B = CF (%*w*/*w*), C = EA (%*w*/*w*), D = external curcumin (%*w*/*w*), and E = sodium hyaluronate (NaHA) (%*w*/*w*). To assess the predictability of the regression model, all models obtained were validated. The predictive root mean square error (predictive RMSE) and predictive r^2^ of all regression models calculated using Equations (1)–(5) are shown in Table 2.

Predicted r^2^ close to one and low predictive RMSE should be obtained for the model with good predictability. In this study, it was found that regression models for z-average, d_50_, and d_90_ had predicted r^2^ higher than 0.9 (0.9120–0.9992), and the predictive RMSEs of these models were 12.00, 26.30, and 88.24, respectively. This indicates the good predictability of these models. However, the predictabilities of models for curcumin content showed low power (r^2^ of 0.8673 and 0.7140 for CM content and %LA of CM, respectively).

**Figure 3:**
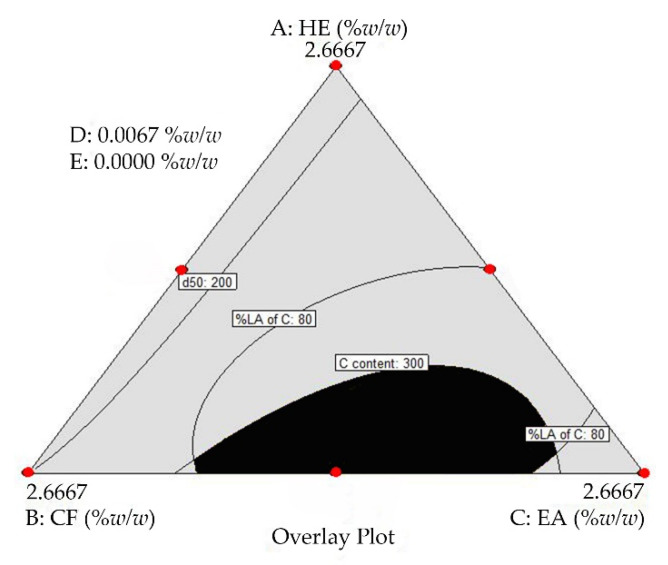
Overlay plot. Black area = the optimal region; A = HE; B = CF; C = EA; D = external curcumin; and E = sodium hyaluronate (NaHA). Lines represent the TE-NPs formulations with minimum and maximum dependent variable values according to the acceptance limit. The spots (🔴) represent the design points.

The optimal TE-NPs formulation was selected from the optimal region (Figure 3). To determine the optimal region, the acceptance limits of desired dependent variables were specified first. The highest curcumin content obtained in MPV design was 348.67 µM. Thus, the acceptance lower limit of curcumin content of 300 µM was used. The acceptance limit of 80–120% LA for curcumin was chosen [62]. Particle size plays a crucial role in the delivery of nanoparticles to tumor cells. The nanoparticles of appropriate size can be selectively delivered to tumor cells and can escape from the defensive system of body. Angiogenesis in cancer cells results in abnormalities—namely, hypervascularization, aberrant vascular architecture, extensive production of vascular permeability factors stimulating extravasation within tumor tissues, and lack of lymphatic drainage. It allows the passive accumulation of the nanoparticles in tumor tissue, which is known as an enhanced permeability and retention effect (EPR effect) [63,64,65,66]. To achieve the extravasation into a tumor by the EPR effect, nanoparticles’ size should be below 200 nm [67]. Moreover, nanoparticles must have an appropriate circulation half-life, avoiding the action of the mononuclear phagocyte system (MPS) and the reticuloendothelial system (RES). To overcome these effects, the nanoparticles’ size must not exceed 400 nm to escape from the MPS effect [67]. Thus, the acceptance upper limit of 200 nm for z-average and d_50_, and 400 nm for d_90_ were specified in this study. The optimal region (black area) that was obtained by overlaying between dependent variable plots is shown in Figure 3. Each individual point in this optimal region represents an appropriate TE-NPs formulation. In this study, the optimal TE-NPs formulation consisting of 1.3697 %*w*/*w* CF, 1.2970 %*w*/*w* EA, and 0.0067 %*w*/*w* external curcumin was selected. The physicochemical properties of the optimal TE-NPs formulations are shown in Table 3.

**Figure 4:**
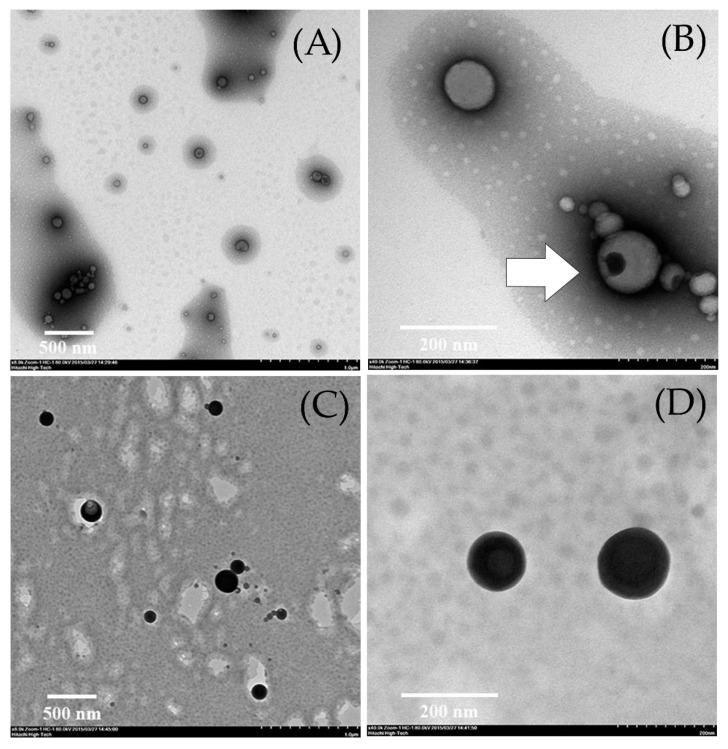
The particle morphology of turmeric rhizome extract nanoparticles of CT_OP_ formulation observed by TEM. (**A**,**B**) = sample coated with 2% uranyl acetate: 8000× and 40,000×, respectively; (**C**,**D**) = sample coated with 2% osmium tetroxide: 8000× and 40,000×, respectively.

It was found that the optimal TE-NPs had physicochemical properties within the acceptance limits. The optimal TE-NPs were stable for up to 3 months when stored at 5 °C. Furthermore, the results show that the optimal TE-NPs had curcumin content higher than TE-NPs prepared from the ethanol extract (EV) obtained directly from turmeric rhizome powder extraction. The TEM study confirmed that the optimal TE-NPs had a spherical shape with a size below 200 nm (Figure 4). Additionally, it was noticed that the TE-NPs nanoparticles had a special structure that looked like a polyp inside the particle (Figure 4B, white arrow). This special structure may be the agglomeration of the solid particles containing the turmeric extract. The result of this study shows that stable TE-NPs formulation containing increased curcumin content was successfully developed.

### 2.3. Cytotoxicity of Turmeric Rhizome Extract Nanoparticles in HepG2 Cells

**Figure 5:**
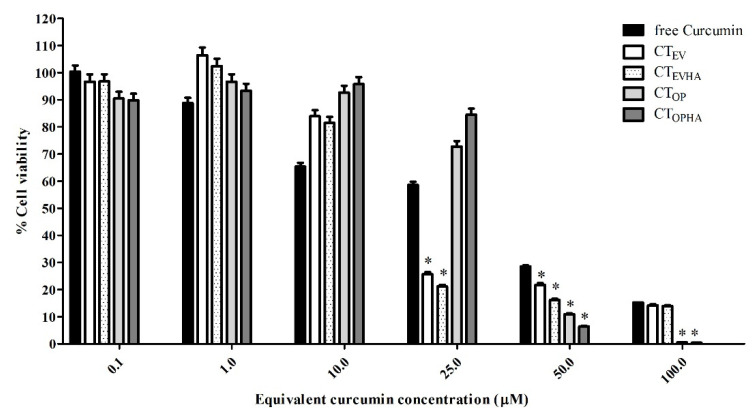
Cytotoxicity of turmeric rhizome extract nanoparticles quantified by MTT assay. The data are expressed as the percentage relative to the vehicle (control) and are shown as mean ± SD (*n* = 3). CT_EVHA_ and CT_EV_ = TE-NPs prepared from ethanol extract (EV) with and without sodium hyaluronate (NaHA), respectively. CT_OPHA_ and CT_OP_ = TE-NPs prepared from optimal formulation with and without sodium hyaluronate (NaHA), respectively. *, *p* < 0.05 versus free curcumin.

The cytotoxicity of TE-NPs compared with free curcumin is shown in Table 4 and Figure 5. It was found that free curcumin and four TE-NPs exhibited a cytotoxicity effect on the human hepatoma HepG2 cells. The IC50 values were 43, 40, 37, 41, and 42 μM for free curcumin, CT_EV_, CT_EVHA_, CT_OP_, and CT_OPHA_, respectively. Although, the IC50 values for TE-NPs were shown to be slightly lower than those of free curcumin, all TE-NPs formulations showed a significantly stronger inhibition effect than free curcumin at the equivalent curcumin concentrations of 50–100 µM (*p*-value < 0.05). The higher inhibition effect of TE-NPs might be due to other compositions contained in the turmeric rhizome in addition to curcumin. These compositions were DCM, BDCM, and turmerone compounds [6,7,9]. Ethanol was also reported to have inhibition effect on HepG2 cells, with an IC50 value of 3.13 %*v*/*v* [68,69,70,71,72]. In this experiment, ethanol concentrations of TE-NPs samples were in the range of 0.001–1.518 %*v*/*v* (for CT_EV_ and CT_EVHA_) and 0.001–0.983 %*v*/*v* (for CT_OP_ and CT_OPHA_), depending on the equivalent curcumin concentration in each formulation. These maximum levels of ethanol contained in TE-NPs samples were 2–3 times lower than IC50. Therefore, it can be assumed that ethanol has a negligible inhibition effect on HepG2 cells. In addition, it was shown that the treatment using the developed optimal formulations (CT_OP_, and CT_OPHA_) at equivalent curcumin concentration of 50–100 µM inhibited the proliferation of HepG2 better than CT_EV_ and CT_EVHA_ (*p* value < 0.05).

To investigate the enhancing effect of sodium hyaluronate, TE-NPs formulations with sodium hyaluronate coating were developed and tested for their inhibitory effect to HepG2 cells. The results show that for CT_EVHA_ and CT_OPHA_ formulations, at 50 µM equivalent curcumin concentration, the sodium hyaluronate coated TE-NPs formulations showed significantly higher inhibitory effects than the uncoated formulations (*p* value < 0.05). This indicates that sodium hyaluronate coating may enhance the inhibition effect of TE-NPs toward HepG2 cells. This enhancing effect is dose-dependent.

## 3. Materials and Methods

### 3.1. Materials

Turmeric (*Curcuma longa* Linn., Zingiberaceae) rhizome powder was purchased from a medicinal herb store in Bangkok, Thailand. Plant sample was identified by Dr. Pongtip Sithisarn, Department of Pharmacognosy, Faculty of Pharmacy, Mahidol University, Bangkok, Thailand. Curcumin 98% was purchased from AK Scientific, Union city, CA, USA. Turmeric oil was purchased Thai-China Flavours and Fragrances Industry Co. Ltd., Phra Nakhon Si Ayutthaya, Thailand. Sodium hyaluronate was purchased from Bloomage Freda Biopharm, Jinan, China. Curcumin, desmethoxycurcumin, and bisdesmethoxycurcumin standards were purchased from USP, Rockville, MD, USA. Analytical HPLC grade acetonitrile was purchased from Scharlab S.L., Barcelona, Spain. Absolute ethanol, hexane, chloroform, and n-butanol were purchased from RCI Labscan Limited, Bangkok, Thailand. Ethyl acetate was purchased from J.T. Baker, Phillipsburg, NJ, USA. The 95% ethanol was purchased from The Liquor Distillery Organization, Chachoengsao, Thailand. Water for Injection was purchased from A.N.B. Laboratories Co., Ltd., Bangkok, Thailand. Benzene was purchased from Panreac quimica SA, Barcelona, Spain. Dimethyl sulfoxide (DMSO) ≥99.5%, 3-[4,5-dimethylthiazole-2-yl]-2,5-diphenyl tetrazolium bromide (MTT) dye, phosphomolybdic acid hydrate, and Dulbecco’s Modified Eagle Medium (DMEM) were purchased from Sigma-Aldrich, St. Louis, MO, USA. Phosphate-buffered solution pH 7.4 and fetal bovine serum (FBS) were purchased from JR Scientific, Inc., Woodland, CA, USA. Penicillin streptomycin solution was purchased from Life Technologies, Carlsbad, CA, USA.

### 3.2. Preparation of Turmeric Rhizome Fraction

Turmeric powder (200 g) was mixed with 95% ethanol (600 g). After being kept at room temperature for 48 h, the mixture was filtered through filter paper (Whatman no. 2) and nylon filter pore size 0.45 μm, consecutively. The filtrate was dried at 50 °C. The ethanol extract (EV) was dispersed in water with weight to volume ratio of EV/water of 1:10. The mixture was sonicated for 10 min. Solvent–solvent extraction process was conducted using four solvents including hexane, chloroform, ethyl acetate, and n-butanol. The ratio of solvent/EV aqueous dispersion used was 1:1 by volume. First, hexane was added into EV aqueous dispersion, stirred for 30 min, and left at room temperature until the aqueous phase was completely separated from the hexane phase. Then the hexane phase was withdrawn. Hexane extraction was repeated with the remaining turmeric aqueous dispersion for two times. Three collected parts of hexane phase were combined and dried at 50 °C by using a rotary evaporator model Buchi Rota vapor R200 (BÜCHI Labortechnik AG, Flawil, Switzerland). The remaining turmeric aqueous dispersion was further extracted using chloroform with the extraction procedure exactly the same as that described above for hexane. The remaining turmeric aqueous dispersion was further extracted by using ethyl acetate and then n-butanol, consecutively. The dried turmeric rhizome fractions obtained from solvent extraction—i.e., ethanol extract (EV), hexane fraction (HE), chloroform fraction (CF), ethyl acetate fraction (EA), n-butanol fraction (BU), and the remaining aqueous fraction (AQ)—were weighed and dissolved in 95% ethanol to obtain the final concentration of 2.5 %*w*/*w*. The fractions in ethanol were prepared and kept in a glass bottle with screw cap, stored at 5 °C, and protected from light until use.

### 3.3. Characterization and Curcuminoids Content Analysis of Turmeric Rhizome Extracts

Phytochemical analysis of turmeric rhizome fractions was carried out by using thin layer chromatography (TLC). Five microliters of turmeric rhizome fractions and 0.05 %*w*/*v* standard curcumin, desmethoxycurcumin, and bisdesmethoxycurcumin were separately spotted on a TLC plate (silica gel GF254). Benzene/chloroform/ethanol 49:49:2 by volume was used as a solvent system. The solvent front was 8 cm. After development, the TLC plate was examined under UV at the wavelengths of 254 nm and 366 nm in a UV chamber. Then, the TLC plate was sprayed with 10% phosphomolybdic acid spray reagent and heated at 105 °C for 5 min. The hRf values of the samples were calculated and compared with hRf values of the standards and hRf values specified in Thai Herbal Pharmacopoeia 2020, Volume 1 [56].

Curcuminoid content of turmeric rhizome fractions (curcumin, desmethoxycurcumin, and bisdesmethoxycurcumin) were quantitatively analyzed using a modified HPLC method developed by Wichitnithad W and coworkers (2009) [57]. A Shimadzu-VP system equipped with a SCL-10A VP controller, a LC-10AD VP pump, a SIL-10AD VP auto- injector, a DGU-14 degasser, an SPD-10A VP UV-VIS detector, and Shimadzu CLASS-VP software (Shimadzo corporation, Kyoto, Japan) were used together with Hypersil GOLD C18 column (250 × 4.6 mm i.d.; 5 µm, Thermo Fisher Scientific Inc., Waltham, MA, USA). A reverse-phase HPLC analysis was carried out by using an isocratic system with 1% acetic acid and acetonitrile at the volume ratio of 61:39 as a mobile phase at a flow rate of 1.2 mL/min. The injection volume was 10 µL and analytical time was 25 min. A detection wavelength of 425 nm was used. The method that was used was validated for accuracy, precision, specificity, linearity, and sensitivity.

### 3.4. Preparation of Turmeric Rhizome Extract Nanoparticles

Turmeric rhizome extract nanoparticles (TE-NPs) were spontaneously formed by the solvent displacement method called the Ouzo effect, which was first named and described by Vitale and Katz in 2003 [47]. The formulations consisted of 2.5 %*w*/*w* turmeric rhizome fraction in ethanol solution, Water for Injection (WFI), external curcumin (extCM), and/or sodium hyaluronate (NaHA). By using a syringe with a 25G needle, turmeric rhizome fraction in ethanol solution at certain quantity by weight was dropped into WFI at the rate of 60 drops per minute. The mixture was continuously stirred at 400 rpm for 10 min. For cases where extCM was used, it was completely dissolved in turmeric rhizome fraction in ethanol solution by sonication for 10 min before subsequent nanoparticles formation. For cases where NaHA was added, 0.1 %*w*/*w* NaHA aqueous solution was dropped into the TE-NPs dispersion. The TE-NPs dispersion was continuously stirred at 400 rpm for 10 min.

### 3.5. Characterization and Curcuminoids Content Analysis of Turmeric Rhizome Extract Nanoparticles

The particle characteristics (z-average, d_50_, d_90_, derived count rate, and PDI) and zeta potential values of TE-NPs were studied by dynamic light scattering technique (DLS) using Zetasizer Nano ZS (Malvern Instruments, Worcestershire, UK). The measurement was set at equilibrated time of 2 min, 173° detection optics backscatter detection, numbers of run 10 times, run duration 10 s, and the measurement was carried out in triplicates. The refractive index required for size measurement was determined by Abbe Refractometer NAR-3T (Atago, Tokyo, Japan) set at 25 °C and the wavelength of 589 nm. The refractive index of turmeric rhizome fraction of 1.38 was used. Curcuminoids content in TE-NPs were quantitatively analyzed using a validated modified HPLC method, described above.

### 3.6. Determination of the Optimal Turmeric Rhizome Extract Nanoparticles Formulation

To determine the optimal TE-NPs formulation, the regression model was constructed by using the mixture–process variables experimental (MPV) design [73]. The quantity of 2.5 %*w*/*w* HE, CF, and EA ethanol solutions were selected as mixture components. The quantity of external curcumin (extCM) and 0.1 %*w*/*w* sodium hyaluronate (NaHA) aqueous solution were selected as process variables. Physicochemical properties of TE-NPs—namely, curcuminoid contents, %LA of curcuminoids, and particle characteristics—were dependent variables. The ranges of actual and coded mixture components and process variables are shown in Table 5 The 47-run MPV design was generated by Design-Expert 9 (Stat-Ease, Inc., Minneapolis, MN, USA) using the best optimal design algorithm with D-optimality criterion. The basis for 47 runs was the 36 terms in the MPV model, 5 extra-points to assess model lack-of-fit, 5 replicated points, and 1 additional center point. TE-NPs were prepared by the method described above.

The physicochemical properties of TE-NPs were measured, and the results were used to construct regression models by ANOVA with backward elimination regression at alpha of 0.05. The predictability of regression models was validated by an external data set of 10 formulations that were not included in the MPV design data set. To demonstrate the predictability, the predictive root mean square error (predictive RMSE) and predictive r^2^ were calculated according to the following equations [74]:

(6) $$\mathrm{predictive}\;\mathrm{RMSE}\;=\sqrt{\frac{\sum {\left(y_{\mathrm{experimental}}-y_{\mathrm{predicted}}\right)}^{2}}{N}}$$

(7) $${\mathrm{predictive}\;r}^{2}=1-\frac{\sum {\left(y_{\mathrm{experimental}}-y_{\mathrm{predicted}}\right)}^{2}}{\sum {\left(y_{\mathrm{experimental}}-y_{\mathrm{mean}}\right)}^{2}}$$

where y_experimental_ is the dependent variable value obtained from the experiment, y_mean_ is an average dependent variable of the results obtained from the experiment, y_predicted_ is the dependent variable value obtained from the regression model, and N is total number of experimental points.

To determine the optimal TE-NPs formulation, the contour plots were constructed with the acceptance ranges of the desired dependent variables and overlayed by Design Expert 9. The overlapping area was an optimal region. Each individual point in this optimal region represented an appropriate formulation. The optimal formulation could be selected from this region.

### 3.7. Preparation, Characterization, and Cytotoxicity Test of the Optimal Turmeric Rhizome Extract Nanoparticles

The optimal turmeric rhizome extract nanoparticles formulation (CT_OP_) obtained from MPV design was prepared by the method described above with aseptic techniques. The physicochemical properties and particle morphology of CT_OP_ were determined. The particle morphology was observed by using a transmission electron microscope (TEM) model Hitachi HT7700 (Hitachi High-Technologies Corporation, Tokyo, Japan) with accelerated voltage preset of 80 kV. Two techniques were used to prepare the sample. First, the TE-NPs dispersion was dropped on a paraffin film. A Formvar-coated copper grid was gently placed on the drop for 2 min, removed, stained by 2% uranyl acetate solution, and left overnight in desiccator before study. Second, the grid was placed on the drop of the TE-NPs dispersion on the paraffin film, removed, and left overnight in desiccator saturated with vapor of osmium tetroxide before study.

The cytotoxicity test using MTT assay was carried out for the optimal TE-NPs formulation in comparison with free curcumin and the selected TE-NPs formulations.

#### 3.7.1. Cell Culture

Human hepatoma (HepG2) cells (catalog number HB-8065 from American Type Culture Collection; ATCC) were maintained in Dulbecco’s Modified Eagle Medium (DMEM) supplemented with 10% fetal bovine serum (FBS) and 1:100 penicillin/streptomycin (10,000 units/mL) at 37 °C and 5% CO_2_.

#### 3.7.2. MTT Assay (Cytotoxicity Assay)

HepG2 cells (1 × 10^4^ cells/well) were cultured in 96-well plates, in 200 µL of DMEM supplemented with 10% FBS and 1% streptomycin/penicillin solution (Gibco) and incubated at 37 °C and 5% CO_2_ humidified atmosphere for overnight as previously described [75]. Cells were treated with solubilized free curcumin in dimethyl sulfoxide (DMSO) and TE-NPs at the final equivalent curcumin concentrations of 0.1, 1, 10, 25, 50, and 100 µM. DMSO 1 %*v*/*v* was used as a control. This study was performed in triplicate, with two replicate wells. The relative number of viable cells was determined after 24 h incubation by adding 1 mg/mL of 3-[4,5-dimethylthiazol-2-yl]-2,5-diphenyl tetrazolium bromide (MTT) and incubating the cells for a further 4 h. The formazan crystals formed were dissolved with DMSO. The absorbance values of the solution at wavelength of 570 nm, which was directly relative to the viable cells, were determined using an Infinite M200 microplate reader (Tecan Sales Austria GmbH, Grödig, Austria). The percentage of cell viability was calculated as follows:

(8) $$\%\;\mathrm{Cell}\;\mathrm{viability}\;=\frac{\mathrm{Absorbance}\;\mathrm{of}\;\mathrm{treated}\;\mathrm{cells}}{\mathrm{Absorbance}\;\mathrm{of}\;\mathrm{control}\;\mathrm{cells}}\times 100$$

### 3.8. Statistical Analysis

The data obtained are expressed as mean ± standard deviation (SD) of triplicates. Unpaired t-test or one-way ANOVA was used to compare means (α = 0.05). All analyses were performed using PASW Statistics for Windows, version 18.0 (SPSS Inc., Chicago, IL, USA).

## 4. Conclusions

Nanoparticles were successfully prepared from turmeric rhizome fractions in this study. By applying 47-run D-optimal mixture–process variables experimental design, the appropriate formulation of stable nanoparticles was obtained. The optimal TE-NPs formulation had physicochemical properties within the acceptance limits after a 3-month storage period. In addition, regression models with good predictability for three desired dependent variables including z-average, d_50_, and d_90_ could be determined. The results from a cytotoxicity study using human hepatoma HepG2 cells show that the optimal TE-NPs had stronger cytotoxic effects than free curcumin. Thus, optimal TE-NPs could be successfully developed by using the mixture–process variables experimental design. It was found that the aqueous solubility of curcumin was increased in the optimal formulation system. Moreover, the addition of curcumin in the TE-NPs formulation might be applicable for cases where there is a biological variation of curcuminoids content. It should be noted that an inhibition effect of TE-NPs was found in an in vitro experiment using HepG2 cells. Further clinical study should be performed to confirm this result.

## Figures and Tables

**Table 1:** Curcuminoids content of turmeric rhizome fractions analyzed by HPLC (Mean ± SD, *n* = 3).

Fraction	Curcuminoids Content (mg/g of Dried Extract)			
	CM	DCM	BDCM	Total Curcuminoids
EV	147.97 ± 1.24	68.64 ± 0.57	69.39 ± 0.55	285.99 ± 2.35
HE	2.21 ± 0.02	1.12 ± 0.01	1.54 ± 0.01	4.88 ± 0.04
CF	114.05 ± 1.59	52.26 ± 0.75	13.72 ± 0.13	180.04 ± 2.47
EA	132.09 ± 1.82	88.42 ± 1.31	200.89 ± 3.77	421.41 ± 6.84
BU	4.38 ± 1.82	4.18 ± 1.74	3.58 ± 1.50	12.14 ± 5.06
AQ	ND	ND	ND	ND

CM = curcumin; DCM = desmethoxycurcumin; BDCM = bisdesmethoxycurcumin; EV = ethanol extract; HE = hexane fraction; CF = chloroform fraction; EA = ethyl acetate fraction; BU = n-butanol fraction; AQ = aqueous fraction; ND = cannot be detected.

**Table 2:** The predictive root mean square error (predictive RMSE) and predictive r^2^ of all regression model equations.

Regression Model	Min	Max	r^2^	Predicted r^2^	RMSE	Predicted RMSE
CM content (Y_m1_)	0.00 ± 0.00	348.67 ± 6.08	0.9603	0.8673	1.14	49.11
%LA of CM (Y_m__2_)	0.00 ± 0.00	112.02 ± 9.71	0.9480	0.7140	0.75	17.38
Z-average (Y_m__5_)	144.5 ± 1.3	281.3 ± 4.4	0.9635	0.9120	8.00	12.00
d_50_ (Y_m__6_)	152.3 ± 0.6	477.3 ± 25.2	0.9003	0.9891	0.97	26.30
d_90_ (Y_m__7_)	238.7 ± 7.1	989.7 ± 151.5	0.8961	0.9992	1.80	88.24

**Table 3:** Physicochemical properties of the optimal turmeric rhizome extract nanoparticles stored at 5 °C for 3 months (Mean ± SD, *n* = 3).

Dependent Variables	Acceptance Limit	Initial	3 Months
CM content (µM)	≥300 µM	357.48 ± 8.39	358.84 ± 4.65
%LA of CM (%LA)	80–120 %LA	92.74 ± 2.18	93.09 ± 1.21
Z-average (nm)	≤200 nm	159.6 ± 1.7	166.6 ± 0.6
d_50_ (nm)	≤200 nm	169.7 ± 2.1	177.0 ± 1.0
d_90_ (nm)	≤400 nm	272.3 ± 9.1	276.7 ± 2.5

CM = curcumin.

**Table 4:** Cytotoxicity study of selected turmeric rhizome extract nanoparticles by MTT assay (Mean ± SD, *n* = 3).

Curcumin Concentration (µM)	% Cell Viability				
	Free Curcumin	CTEV	CTEVHA	CTOP	CTOPHA
0.1	100.34 ± 2.31	96.70 ± 2.62	96.76 ± 2.62	90.49 ± 2.45	89.82 ± 2.43
1.0	88.80 ± 1.99	106.38 ± 2.88	102.40 ± 2.78	96.74 ± 2.62	93.34 ± 2.53
10.0	65.39 ± 1.36	83.92 ± 2.27	81.47 ± 2.21	92.63 ± 2.51	95.76 ± 2.60
25.0	58.61 ± 1.17	25.69 ± 0.70	21.19 ± 0.57	72.77 ± 1.97	84.49 ± 2.29
50.0	28.55 ± 0.36	21.78 ± 0.59	16.17 ± 0.44	10.95 ± 0.30	6.45 ± 0.17
100.0	15.28 ± 0.00	14.18 ± 0.38	13.92 ± 0.38	0.60 ± 0.02	0.43 ± 0.01

**Table 5:** Variables used in MPV design.

Variables	Actual Variables			Coded Variables	
	Unit	Low	High	Low	High
Mixture components					
2.5 %*w*/*w* HE	%*w*/*w*	0	2.6667	0	1
2.5 %*w*/*w* CF	%*w*/*w*	0	2.6667	0	1
2.5 %*w*/*w* EA	%*w*/*w*	0	2.6667	0	1
Process variables					
External CM	%*w*/*w*	0	0.0067	−1	1
0.1 %*w*/*w* NaHA	%*w*/*w*	0	3.3333	−1	1

HE = hexane fraction; CF = chloroform fraction; EA = ethyl acetate fraction; CM = curcumin; and NaHA = sodium hyaluronate.

## Data Availability

The data presented in this study are available in the article.

## Supplementary Materials

The following supporting information can be downloaded online, Table S1: Physicochemical properties of turmeric rhizome extract nanoparticles formulation obtained from mixture―process variables experimental design.

## Sample Availability

Samples of the compounds are not available from the authors.
