# Supplementary material for: Design of Turmeric Rhizome Extract Nano-Formula for Delivery to Cancer Cells
Source: Molecules. 2022 Jan 28;27(3):896. doi: 10.3390/molecules27030896 (PMC8838412; doi:10.3390/molecules27030896)
Supplement: Supplementary file 1 [file molecules-27-00896-s001.zip › molecules-1540051-supplementary.pdf]

*Article*

# Design of turmeric rhizome extract nano- formula for delivery to cancer cells

**Table S1.** Physicochemical properties of turmeric rhizome extract nanoparticles formulation obtained from mixture-process variables experimental design.

| Formulation | $Y_{m1}$ | $Y_{m2}$ | $Y_{m3}$ | $Y_{m4}$ | $Y_{m5}$ | $Y_{m6}$ | $Y_{m7}$ | $Y_{m8}$ |
|---|---|---|---|---|---|---|---|---|
| M01 | 77.28 | 66.24 | 54.38 | 35.08 | 160.4 | 171.0 | 285.3 | 0.150 |
| M02 | 1.81 | 1.03 | 0.00 | 0.00 | 182.1 | 200.0 | 363.3 | 0.161 |
| M03 | 150.41 | 71.61 | 65.88 | 54.40 | 171.2 | 223.0 | 452.3 | 0.302 |
| M04 | 181.49 | 63.77 | 57.93 | 49.05 | 187.2 | 238.0 | 526.3 | 0.329 |
| M05 | 7.95 | 68.96 | 60.77 | 45.39 | 205.5 | 298.0 | 645.3 | 0.490 |
| M06 | 86.93 | 65.91 | 66.53 | 68.06 | 165.2 | 178.3 | 280.3 | 0.124 |
| M07 | 38.89 | 58.69 | 60.92 | 66.12 | 200.8 | 293.0 | 561.3 | 0.416 |
| M08 | 9.08 | 40.10 | 59.44 | 70.01 | 255.8 | 476.7 | 926.3 | 0.571 |
| M09 | 0.00 | 0.00 | 0.00 | 0.00 | 191.3 | 212.7 | 368.3 | 0.161 |
| M10 | 89.65 | 93.46 | 80.13 | 75.51 | 163.6 | 178.0 | 304.3 | 0.154 |
| M11 | 83.87 | 60.11 | 38.35 | 18.18 | 197.4 | 249.7 | 924.7 | 0.442 |
| M12 | 5.22 | 30.51 | 45.28 | 68.15 | 253.9 | 395.0 | 766.0 | 0.535 |
| M13 | 52.39 | 68.55 | 42.50 | 19.29 | 196.7 | 280.0 | 612.0 | 0.442 |
| M14 | 1.77 | 1.04 | 0.00 | 0.00 | 281.3 | 477.3 | 985.0 | 0.572 |
| M15 | 76.78 | 21.43 | 36.60 | 53.73 | 232.3 | 395.7 | 871.7 | 0.601 |
| M16 | 221.63 | 55.13 | 48.47 | 28.40 | 167.9 | 177.7 | 292.3 | 0.152 |
| M17 | 134.01 | 70.09 | 75.02 | 73.84 | 162.5 | 173.0 | 262.7 | 0.113 |
| M18 | 306.06 | 81.87 | 85.17 | 85.48 | 198.5 | 298.3 | 654.0 | 0.477 |
| M19 | 0.41 | 10.86 | 0.00 | 0.00 | 272.3 | 444.3 | 924.7 | 0.556 |
| M20 | 214.68 | 55.13 | 42.63 | 21.77 | 201.5 | 305.0 | 637.0 | 0.469 |
| M21 | 173.88 | 88.10 | 89.58 | 86.21 | 160.6 | 172.3 | 268.0 | 0.113 |
| M22 | 3.91 | 4.48 | 0.00 | 0.00 | 275.1 | 469.3 | 957.3 | 0.570 |
| M23 | 348.67 | 90.35 | 87.43 | 84.52 | 154.9 | 164.3 | 251.3 | 0.105 |
| M24 | 180.27 | 64.35 | 62.88 | 67.28 | 218.4 | 319.3 | 708.3 | 0.463 |
| M25 | 121.35 | 44.44 | 50.21 | 66.45 | 175.9 | 190.0 | 312.3 | 0.140 |
| M26 | 141.81 | 61.79 | 39.76 | 18.35 | 158.2 | 171.7 | 277.3 | 0.136 |
| M27 | 174.35 | 81.69 | 73.15 | 59.58 | 147.1 | 156.7 | 244.3 | 0.106 |
| M28 | 169.80 | 82.13 | 78.22 | 70.65 | 191.9 | 293.0 | 558.0 | 0.477 |
| M29 | 174.91 | 47.29 | 60.62 | 70.71 | 162.5 | 175.0 | 270.3 | 0.118 |
| M30 | 36.68 | 13.86 | 27.38 | 53.74 | 260.8 | 431.7 | 989.7 | 0.554 |
| M31 | 62.81 | 62.43 | 78.65 | 107.70 | 174.3 | 188.7 | 304.7 | 0.153 |
| M32 | 253.11 | 87.53 | 77.95 | 76.87 | 169.6 | 183.3 | 308.0 | 0.150 |
| M33 | 182.67 | 58.79 | 39.17 | 19.21 | 180.7 | 223.7 | 530.3 | 0.282 |
| M34 | 280.81 | 95.22 | 88.46 | 90.77 | 170.2 | 215.3 | 402.3 | 0.283 |
| M35 | 0.00 | 0.00 | 0.00 | 0.00 | 231.7 | 322.3 | 619.3 | 0.360 |
| M36 | 42.98 | 23.37 | 40.70 | 62.65 | 212.2 | 322.0 | 671.7 | 0.452 |
| M37 | 253.35 | 112.02 | 98.40 | 89.50 | 187.4 | 242.3 | 462.3 | 0.303 |
| M38 | 81.49 | 22.39 | 34.10 | 52.70 | 207.4 | 268.0 | 535.0 | 0.345 |
| M39 | 1.94 | 2.17 | 0.00 | 0.00 | 203.1 | 252.0 | 509.3 | 0.271 |
| M40 | 58.97 | 51.69 | 70.63 | 82.64 | 219.3 | 337.7 | 769.7 | 0.522 |
| M41 | 38.79 | 19.56 | 39.56 | 66.21 | 226.7 | 340.0 | 748.3 | 0.494 |

| | | | | | | | | |
|---|---|---|---|---|---|---|---|---|
| M42 | 205.83 | 69.20 | 72.95 | 81.77 | 172.1 | 198.0 | 400.3 | 0.234 |
| M43 | 183.30 | 59.93 | 38.80 | 18.39 | 204.2 | 299.3 | 615.0 | 0.455 |
| M44 | 170.12 | 81.00 | 69.33 | 53.16 | 163.7 | 207.3 | 410.3 | 0.285 |
| M45 | 32.72 | 18.07 | 35.80 | 64.46 | 263.1 | 404.3 | 891.0 | 0.537 |
| M46 | 162.42 | 76.10 | 66.83 | 50.36 | 144.5 | 152.3 | 238.7 | 0.104 |
| M47 | 142.33 | 62.02 | 39.28 | 18.24 | 174.1 | 184.7 | 326.7 | 0.149 |
| Min | 0.00 | 0.00 | 0.00 | 0.00 | 144.5 | 152.3 | 238.7 | 0.104 |
| Max | 348.67 | 112.02 | 98.40 | 107.70 | 281.3 | 477.3 | 989.7 | 0.601 |

$Y_{m1}$ = curcumin content (μM), $Y_{m2}$ = % label amount of curcumin (%LA), $Y_{m3}$ = % label amount of desmethoxycurcumin (%LA), $Y_{m4}$ = % label amount of bisdesmethoxycurcumin (%LA), $Y_{m5}$ = z-average (nm), $Y_{m6}$ = $d_{50}$ (nm), $Y_{m7}$ = $d_{90}$ (nm), and $Y_{m8}$ = Polydispersible index (PDI). Underlined values represent the designed formulation with minimum or maximum dependent variable values.
